# Mutation site and context dependent effects of ESR1 mutation in genome-edited breast cancer cell models

**DOI:** 10.1186/s13058-017-0851-4

**Published:** 2017-05-23

**Authors:** Amir Bahreini, Zheqi Li, Peilu Wang, Kevin M. Levine, Nilgun Tasdemir, Lan Cao, Hazel M. Weir, Shannon L. Puhalla, Nancy E. Davidson, Andrew M. Stern, David Chu, Ben Ho Park, Adrian V. Lee, Steffi Oesterreich

**Affiliations:** 1 Department of Human Genetics, University of Pittsburgh, Pittsburgh, PA USA; 2 Department of Pharmacology and Chemical Biology, University of Pittsburgh, Pittsburgh, PA USA; 3 Womens Cancer Research Center, University of Pittsburgh Cancer Institute and Magee-Women Research Institute, Pittsburgh, PA USA; 4 School of Medicine, Tsinghua University, Beijing, China; 5 Department of Pathology, University of Pittsburgh, and MSTP Program, Pittsburgh, PA USA; 6 Central South University Xiangya School of Medicine, Changsha, China; 7 Oncology iMed, AstraZeneca, Alderley Park, Macclesfield, UK; 8 Department of Medicine, Division of Hematology/Oncology, University of Pittsburgh, Pittsburgh, PA USA; 9 Drug Discovery Institute, University of Pittsburgh, Pittsburgh, PA USA; 10 The Sidney Kimmel Comprehensive Cancer Center, The Johns Hopkins University School of Medicine, Baltimore, MD USA; 11 Fred Hutchinson Cancer Research Center and University of Washington, Seattle, WA USA

**Keywords:** *ESR1* mutations, Genome-edited cells, Metastatic breast cancer, Endocrine resistance, RNA-seq

## Abstract

**Background:**

Mutations in the estrogen receptor alpha (ERα) 1 gene (*ESR1*) are frequently detected in ER+ metastatic breast cancer, and there is increasing evidence that these mutations confer endocrine resistance in breast cancer patients with advanced disease. However, their functional role is not well-understood, at least in part due to a lack of *ESR1* mutant models. Here, we describe the generation and characterization of genome-edited T47D and MCF7 breast cancer cell lines with the two most common *ESR1* mutations, Y537S and D538G.

**Methods:**

Genome editing was performed using CRISPR and adeno-associated virus (AAV) technologies to knock-in *ESR1* mutations into T47D and MCF7 cell lines, respectively. Various techniques were utilized to assess the activity of mutant ER, including transactivation, growth and chromatin-immunoprecipitation (ChIP) assays. The level of endocrine resistance was tested in mutant cells using a number of selective estrogen receptor modulators (SERMs) and degraders (SERDs). RNA sequencing (RNA-seq) was employed to study gene targets of mutant ER.

**Results:**

Cells with *ESR1* mutations displayed ligand-independent ER activity, and were resistant to several SERMs and SERDs, with cell line and mutation-specific differences with respect to magnitude of effect. The SERD AZ9496 showed increased efficacy compared to other drugs tested. Wild-type and mutant cell co-cultures demonstrated a unique evolution of mutant cells under estrogen deprivation and tamoxifen treatment. Transcriptome analysis confirmed ligand-independent regulation of ERα target genes by mutant ERα, but also identified novel target genes, some of which are involved in metastasis-associated phenotypes. Despite significant overlap in the ligand-independent genes between Y537S and D538G, the number of mutant ERα-target genes shared between the two cell lines was limited, suggesting context-dependent activity of the mutant receptor. Some genes and phenotypes were unique to one mutation within a given cell line, suggesting a mutation-specific effect.

**Conclusions:**

Taken together, *ESR1* mutations in genome-edited breast cancer cell lines confer ligand-independent growth and endocrine resistance. These biologically relevant models can be used for further mechanistic and translational studies, including context-specific and mutation site-specific analysis of the *ESR1* mutations.

**Electronic supplementary material:**

The online version of this article (doi:10.1186/s13058-017-0851-4) contains supplementary material, which is available to authorized users.

## Background

Gain-of-function mutations in *ESR1* are likely to play a key role in conferring endocrine therapy resistance in 20–40% of estrogen receptor-positive (ER+) metastatic breast cancer, as reviewed in other papers [1–3]. The majority of mechanistic studies have employed overexpression approaches, and results show that the mutant receptors cause ligand-independent growth and decreased sensitivity to antiestrogens [4–8]. Reporter assays and gene expression analysis in transfected cell lines reveal ligand-independent activity of ER, associated with increased expression of classical ER target genes and some novel ER target genes [4–8].

Two recent reports confirmed the ligand-independent activity of mutants in CRISPR generated cell lines [9, 10]. Harrod et al. generated a single Y537S MCF7 clone, in which ER was able to bind to DNA and regulate endogenous targets in a ligand-independent manner [9]. The study also showed that CDK7 is a promising target in *ESR1* mutant, endocrine-resistant disease. The study from Mao et al. focused on the potential role of increased unfolded protein response in ESR1 mutant cells, and the interaction with progestins [10].

We set out to generate the two most frequently identified *ESR1* mutations Y537S and D538G in two ER+ breast cancer cell lines, T47D and MCF-7. Using multiple clones, we performed in-depth functional analysis that confirmed and expanded previous observations, and importantly identified mutation-specific and cell line-specific phenotypes, suggesting the need for the study of the individual mutations in a context-dependent manner. The genome-wide expression data and the models will be excellent resources for the research community studying endocrine resistance caused by ESR1 mutations.

## Methods

### Cell culture

T47D cells were obtained from the American Type Culture Collection/National Cancer Institute (ATCC/NCI) Breast Cancer SPORE program, and MCF7 cells were purchased from the ATCC. Both cell lines were authenticated at the University of Arizona Genetics Core. T47D and MCF7 cells were maintained in RPMI 1640 medium + 10% FBS and DMEM + 5% FBS, respectively. For hormone treatment experiments, cells were deprived in phenol-red-free IMEM with 10% and 5% CSS for T47D and MCF7, respectively. CSS was purchased from Hyclone (#SH30068) and Gibco (#12676). 17β-estradiol (E2) and 4-hydroxytamoxifen (4OHT) were obtained from Sigma, and fulvestrant (Ful) and raloxifene were purchased from Tocris. AZD9496 recently described in Weir et al. [11] was kindly provided by AstraZeneca.

### Generation of genome-edited ESR1 mutant cell lines

**Fig. 1:**
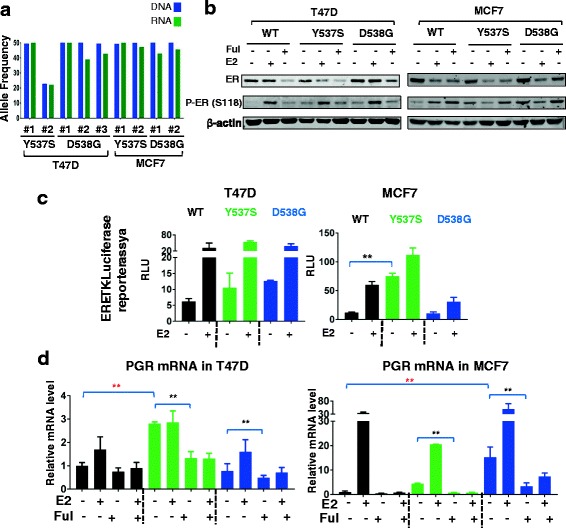
Generation and characterization of *ESR1* mutant, genome-edited MCF7 and T47D cell line models. **a** *ESR1* mutation allele frequency in DNA and RNA was determined by digital droplet PCR. **b** T47D and MCF7 wild-type (*WT*) or mutant clones were pooled and treated with vehicle, 1 nM estradiol (*E2*) or 1 μM of fulvestrant (*Ful*) for 24 h, and lysates were immunoblotted as indicated. The blot is representative of three independent experiments. *ER* estrogen receptor. **c** T47D and MCF7 clones were pooled after hormone deprivation, transfected with ERE-TK, and relative light units (*RLU*) were determined (one-way analysis of variance (Anova), **p < 0.01). The experiment was repeated three times and the figure shows one representative experiment with two biological replicates. **d** Hormone-deprived T47D and MCF7 cells were treated with vehicle, 1 nM E2, 1 μM fulvestrant or 1 nM E2 with 1 μM fulvestrant for 12 h, and RNA was isolated, and RT-qPCR was performed (one-way Anova for comparison of basal level, Student’s *t* test for comparison of fulvestrant response in the presence of E2, *p < 0.05, **p < 0.01)

To select subgenomic RNAs (sgRNAs) (Additional file 1: Table S1) for CRISPR-Cas9 genome-editing of T47D cells [12–15], we utilized a web tool (http://crispr.mit.edu) entering the sequence flanking Y537S and D538G mutations. The oligos were cloned into PX458 (www.addgene.com), also coding for Cas9, tracrRNA, green fluorescent protein (GFP), and the resulting plasmid was transfected along with the respective double-stranded 70 bp oligos into T47D cells. GFP+ cells were sorted by fluorescence-activated cell sorting (FACS), and the mutation was confirmed by Sanger sequencing (Additional file 2: Figure S1) and digital droplet PCR (ddPCR) using previously described methods [16] (Fig. 1a). We obtained two clones for Y537S, three clones for D538G, and three clones for ESR1 wild-type (WT), which were kept as individual clones, and pooled for experimental studies as indicated.

Gene targeting of *ESR1* in MCF7 cells was carried out using recombinant adeno-associated virus (AAV) technology as previously described [17]. Briefly, *ESR1* was targeted using one AAV vector for both the *ESR1* Y537S and D538G mutations. AAV vectors were generated by ligating WT homology arms into an AAV plasmid backbone (Agilent, La Jolla, CA, USA), and site-directed mutagenesis was utilized to generate the Y537S and D538G mutations within the targeting construct. Virus was prepared by co-transfecting HEK-293 T cells with pHelper, pRC (Agilent) and the respective *ESR1* mutation carrying rAAV targeting plasmid: 10^6^ cells were infected, neomycin-resistant clones were isolated using a modified PCR screening strategy [18], and the cells were then exposed to Cre-expressing recombinant adenovirus to remove the neomycin cassette. Clones were confirmed by Sanger sequencing (Additional file 2: Figure S1), and ddPCR (Fig. 1a). Single-stranded cDNA was generated using the First Strand cDNA Synthesis Kit (Amersham Biosciences). Two clones and a targeted WT control for the *ESR1* exon 10 locus were isolated for each *ESR1* mutation. Primer sequences for PCR amplification, mutagenesis, targeting, and sequencing are shown in the Additional file 1: Table S2.

### Immunoblotting

After 3 days in CSS, 120,000 (MCF7) and 90,000 cells (T47D) were plated into 6-well plates. Cells were treated with vehicle control (veh), 1 nM of estradiol (E2), 100 nM fulvestrant (Ful), or their combination. The cells were lysed with radioimmunoprecipitation assay (RIPA) buffer, were sonicated, and 80 ug of proteins were loaded onto SDS-PAGE gel, and then were transferred onto polyvinylidene fluoride (PVDF) membrane. The blots were immuno-stained using antibodies to ERα (D8H8, Cell Signaling Technology) and phospho- ERα (Ser118) (Signalway Antibody). Immunoblots were repeated twice unless otherwise stated.

### Transcriptional reporter activity of WT and mutant ESR1

*ESR1* WT and mutant cells were transfected with ERE-TK-luc and renilla plasmids, and treated with veh or 1 nM E2 for 24 h, as previously described [19]. The luciferase assay kit (Promega) was used according to the manufacturer’s instructions. Relative light units (RLU) were calculated as the ratio of firefly luciferase activity over Renilla luciferase activity. The experiments were performed in three biological replicates, and one-way analysis of variance (Anova) was performed to test the statistical significance.

### RNA-sequencing (RNA-seq) analysis

Individual *ESR1* WT and mutant T47D and MCF7 clones were hormone-deprived in CSS for 3 days, pooled, and plated in quadruplicates in 6-well plates. The cells were treated with veh or 1 nM E2 for 24 h, RNA was isolated using Qiagen RNeasy kit, and RNA-seq was performed obtaining >15 M reads per sample. Salmon was used for quantification of the transcripts using default options and hg38 genome build as the reference [20]. The genes differentially expressed (DE) between WT and mutants were identified by the DEseq2 package using the contrast option to compare mutants to WT and to calculate the adjusted *p* value and fold change (FC) [21]. Genes with maximum transcripts per million (TPM) <1 across all samples were excluded from further analysis due to low gene expression. R was used for statistical analysis, and for plotting of the heatmaps. The chi-square test was used to assess the statistical significance of overlaps in venn diagrams.

### Growth assays

MCF7 or T47D clones were evenly pooled after 3 days of hormone deprivation in CSS, and plated into 96-well plates using 2500 cells per well (MCF7) or 4000 cells per well (T47D). After 24 h the cells were treated with veh, 1 nM E2, 100 nM Ful, or their combination. The cells were harvested after 0, 2, 4, 6, and 9 days and quantified with the FluoResporter kit (Life Technology) following the manufacturer’s protocol, and the half maximal inhibitory concentration (IC-50) was calculated using the PRISM statistical package. One-way Anova was used to compare the IC-50 values. Fold change at the last day was compared between mutants and WT in the veh settings to measure ligand-independent growth (one-way Anova). All experiments were performed in six biological replicates.

### Chromatin-immunoprecipitation (ChIP) assay

ChIP experiments were performed as previously described [22]. Briefly, hormone-deprived WT and mutant cells were treated with veh or 1 nM E2 for 45 minutes. The immunoprecipitation was performed using ERα (HC-20) and rabbit IgG (sc2027) antibodies (Santa Cruz Biotechnologies). Quantitative (q)PCR was employed to quantify the binding enrichment using the primers shown in Additional file 1: Table S3.

### Statistical analysis

Each experiment was performed with biological and technical replicates, and repeated, as indicated. Multiple statistical tests were used to assess the statistical significance depending on the design of the experiments, and the *p* value was calculated accordingly (*p < 0.05, **p < 0.01).

## Results

### Molecular characterization of *ESR1* mutations

Successful genome editing was confirmed by sequencing multiple clones of Y537S and D538G in T47D and MCF7 cells (Additional file 2: Figure S1). The mutation allele frequency was 50%, reflecting heterozygous targeting in all clones except the T47D Y537S#2 clone in which it was 22%. These frequencies correlated well with mRNA expression of WT and mutant ER (Fig. 1a). At the protein level, the pooled clones showed minimal variation at baseline levels with slightly higher expression of D538G than WT, and Y537S slightly lower in both cell lines (Fig. 1b). Fulvestrant decreased protein levels in all clones, although the residual ER protein levels were higher in D538G. Mutant ER displayed twofold to threefold higher constitutive phosphorylation compared to WT ER in both cell lines (Additional file 2: Figure S2A-B), although not to the level previously observed upon overexpression [8]. E2 treatment inhibited phosphorylation in ESR1-mutant MCF7 cells, which was not observed in T47D mutant cells, again suggesting some cell-line-specific effects of mutant ER. Similar data were obtained when using the individual clones (Additional file 2: Figure S2B).

We then tested ER transcriptional activity using reporter assays, and observed a trend towards increased activity in both T47D mutants, and a significant increase in MCF-7 Y537S cells (Fig. 1c). Expression of *PGR* mRNA, a classical ER target gene, was significantly increased in the absence of ligand in T47D Y537S cells (Fig. 1d), and similar data were observed when measuring expression in individual clones (Additional file 2: Figure S2C). In MCF-7 cells, *PGR* was significantly increased in D538G cells (Fig. 1d, Additional file 2: Figure S2C). Ligand-independent activation of *PGR* was inhibited with Ful, confirming ER-dependency of the effect. Of note, we did not detect significant overexpression of the androgen receptor (Additional file 2: Figure S3), which was recently shown to play a role in endocrine resistance [23]. Collectively, these data show overall utility of the models for studying ligand-independent activity of ER mutants, but also provide some evidence for mutation site and cell context-dependent activities.

### Mutant ER displays resistance to anti-E2/ER ligands

**Fig. 2:**
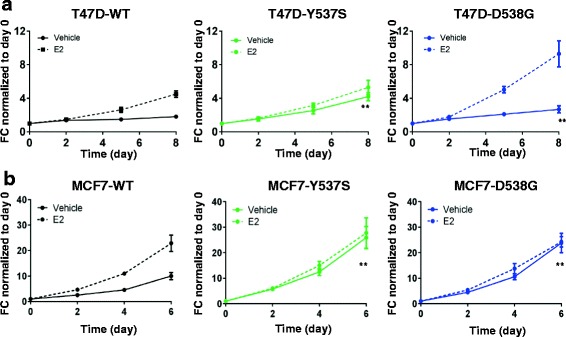
*ESR1* mutant cells exhibit ligand-independent growth. T47D (**a**) and MCF7 (**b**) wild-type (*WT*) or mutant clones were hormone-deprived for 3 days, pooled, treated with vehicle or 1 nM estradiol (*E2*) for up to 8 days, and cell numbers were quantified by the FluoResporter kit. Growth fold change (*FC*) was normalized to day 0: **p < 0.01, one-way analysis of variance, comparison of FC growth between WT and mutant cells on the last day. The experiment was repeated three times with six biological replicates, and similar results were obtained

Y537S and D538G mutant cells showed higher ligand-independent growth compared to WT in both cell lines (Fig. 2). The T47D D538G cells showed an additional strong E2 growth response, not seen in Y537S, or in the MCF7 cells. We had recently reported that growth effects can vary dependent on the source of the CSS [24], and we therefore tested growth in a second CSS lot. We again observed ligand-independent growth of the ER mutant cells, except for T47D-Y537S (Additional file 2: Figure S4), suggesting that there is a factor in serum yet to be identified that contributes to ligand-independent growth, and that varies in CSS lots.

**Fig. 3:**
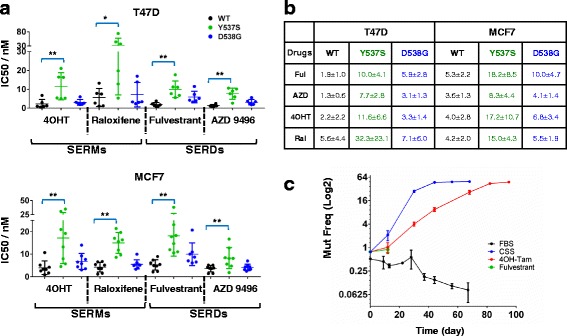
*ESR1* mutant-cells display resistance against selective estrogen receptor modulators (*SERMs*) and selective estrogen receptor degraders (*SERDs*). Graphical (**a**) and tabular (**b**) presentation of half maximal inhibitory concentration (*IC50*) values that were determined in dose–response curves in wild-type (*WT*), Y537S and D538G cells treated with 20 pM estradiol (E2) plus varying doses of 4OHT, raloxifene (*Ral*), fulvestrant (*Ful*), and AZD9496 in T47D and MCF7 cell lines. One-way analysis of variance was performed to compare the IC50 values of mutants to WT within each cell line and drug (*p < 0.05, **p < 0.01). Each *dot* is representative of the mean of a single experiment with six biological replicates. The experiments were performed six times (T47D) or eight times (MCF-7). **c** Pooled T47D-WT and T47D-D538G cells were mixed at a ratio of 99:1 and grown in 10% FBS, 10% CSS, 10% CSS + 1 nM E2 + 100 nM 4OHT, or 10% CSS + 1 nM E2 + 30 nM fulvestrant. The mutation allele frequency was analyzed at each passage using digital droplet PCR

Dose–response studies in 2D growth assays with SERMs and SERDs revealed antiestrogen resistance: cells with mutant ER had higher IC50 for the SERMs 4OHT and raloxifene, and the SERDs fulvestrant and AZD9496 compared to WT (Fig. 3a and b; Additional file 2: Figure S5). We again observed differences between the mutants, with Y537S displaying increased resistance compared to D538G. In addition, AZD9496 was more growth-impeding compared to the other antiestrogens, which was especially obvious in Y537S cells.

Finally, we performed competitive outgrowth experiments in which T47D WT cells were mixed with D538G cells (99:1), and dynamic evolutionary changes were followed by measuring mutant allele frequency using ddPCR (Fig. 3c). In the absence of E2, the mutation frequency of D538G increased until it plateaued at 50% (which represents maximal frequency in the heterozygous D538G clone). A similar competitive advantage of the mutant clone was observed in the presence of 4OHT. In contrast, there was a competitive disadvantage for D538G cells in FBS. In the presence of fulvestrant, all cells died after 2 weeks. Collectively, these data support the previously raised notion [25–27] that SERDs might be more effective against mutant ER compared to SERMs.

### Transcriptome regulation by ER mutants

**Fig. 4:**
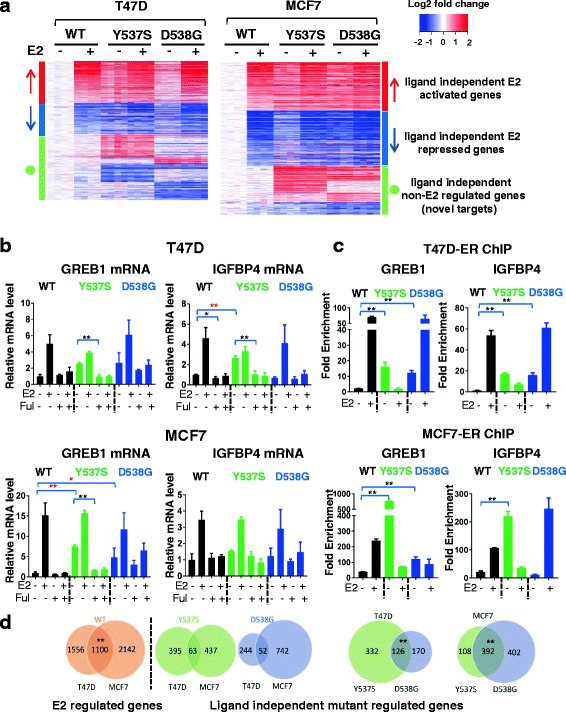
Genome-wide transcriptomic analysis reveals regulation of ligand-independent estrogen receptor (*ER*) targets, and of novel target genes by ERα mutants in T47D and MC7 cells. **a** T47D and MCF7 cell lines were hormone-deprived for 3 days, treated with vehicle (veh) or 1 nM of estradiol (*E2*) for 24 h, RNA was isolated and RNA sequencing analysis was performed. The heat map shows normalized log2 fold change (FC) of genes differentially regulated in mutants vs wild-type (*WT*) in the absence of ligand (FC >2, *p* value <0.005). The genes are sorted based on E2 regulation in WT (*red arrow* ligand-independent E2 activated genes, *blue arrow* ligand-independent E2 downregulated genes, *green circle* ligand-independent non-E2 regulated genes, i.e. “novel target genes”). **b** Hormone-deprived T47D and MCF7 cells were treated with veh, 1 nM E2, 1 μM of fulvestrant (*Ful*) or 1 nM E2 plus 1 μM of Ful for 24 h. RNA was isolated, and *GREB1* or insulin-like growth factor-binding protein 4 (*IGFBP4*) expression was assessed by quantitative RT-qPCR (one-way analysis of variance (Anova) for comparison of basal level, Student’s *t* test for comparison of Ful response in the presence of E2, *p < 0.05, **p < 0.01). The experiment was repeated twice with three biological replicates each time. **c** Cells were hormone-deprived, treated with 1 nM of E2 for 45 minutes, and chromatin-immunoprecipitation (*ChIP*) assays were performed on the ER binding sites on *GREB1* and *IGFBP4* promoters. The data are presented as fold enrichment compared to IgG control (one-way Anova, **p < 0.01). The experiment was repeated twice with three biological replicates each time and the figure shows a representative experiment. **d** The chi-square test was used to assess the statistical significance of overlaps in venn diagrams. *Left panel* overlap of E2-regulated genes in WT cells between the cell lines (chi-square test, **p < 0.01). *Right panel* overlap of ligand-independent target genes between different mutations within each cell line and between the two cell lines (chi-square test, **p < 0.01)

RNA-seq analysis was performed to determine the effect of the mutations on endogenous target gene expression. Analysis of variable genes confirmed that the biological replicates clustered together (Additional file 2: Figure S6), and that the mutants are very different from WT in the vehicle setting (Additional file 2: Figure S7). A total of 1198 and 1327 genes were differentially regulated comparing WT and mutant cells in the absence of ligand in T47D and in MCF7 cells, respectively (FC >2, *p* < 0.005) (Fig. 4a, Additional file 1: Table S4). The majority of the differentially expressed genes were estrogen-regulated in WT clones, supporting the ligand-independent activity of the mutant receptor.

Among the ligand-independent regulated genes were the classic ER target genes *GREB1* and *IGFBP4* (Additional file 1: Table S4, Additional file 2: Figure S9). Ligand-independent expression was confirmed in pooled (Fig. 4b) and in individual (Additional file 2: Figure S8) mutant MCF7 and T47D cells, although we again observed mutation site-specific and cell-line-specific differences in the effects. ChIP analysis revealed increased ER binding to the *GREB1* and *IGBP4* promoters in the absence of ligand in T47D and MCF-7 cells (Fig. 4c). *IGFBP4* transcript levels were not increased significantly in T47D-D538G and MCF7-Y537S despite ER recruitment as observed by ChIP, suggesting that promoter occupancy is not sufficient to initiate transcription. Ability to inhibit the ligand-independent expression with fulvestrant (Fig. 4b), and ESR1 knockdown using small interfering RNA (siRNA) (Additional file 2: Figure S10), confirms ER-dependency of such ligand-independent regulation of expression.

Given our observations of mutation site-specific and cell-line-dependent effects on phenotypes and candidate target genes, we quantified the overlap of ligand-independent target genes between the mutants (within one cell line), and between the cell lines (within one mutant). While there was significant overlap of the ligand-independent target genes when comparing the two mutations (Y537S and D538G) within the individual cell lines (Fig. 4d), there were some unique target genes for both mutants. Despite significant overlap of E2 target genes regulated by WT ER when comparing T47D and MCF7 cells, there was limited overlap when comparing the ER mutant ligand-independent target genes between the two cell lines (Fig. 4d).

The RNA-seq analysis also led to the identification of a set of “novel” target genes (*n* = 425 in MCF7, and *n* = 570 in T47D) that were not E2-regulated in WT cells, but instead were differentially expressed in the *ESR1* mutant clones in the absence of E2 (Fig. 4a). There was significant overlap of these novel target genes between the two mutants within each cell line (*p* value <2E-16), but there was limited overlap between the different cell lines (Additional file 2: Figure S11). Despite the limited overlap, Ingenuity Pathway Analysis (IPA) of the novel genes showed enrichment of metastatic associated phenotypes including “cell movement” (Additional file 1: Table S5). Genes from these pathways will be candidates for future studies when addressing mechanisms for the metastatic propensity of *ESR1* mutant cells.

## Discussion

In this study, we report the generation, characterization and transcriptome analysis of genome-edited “knock-in” models of the most frequent *ESR1* mutations, Y537S and D538G. As recently reported by others [4–8], our data show that the mutant receptors gain ligand-independent transcriptional activity, and this is associated with ligand-independent growth and endocrine resistance. Our study is the first comparing the effect of two mutations, in two different genome-edited breast cancer cell lines, allowing us to conclude that there are mutation-dependent and context-dependent differences.

The majority of previous reports have employed cell lines transfected with ER constructs, potentially resulting in effects associated with non-physiological overexpression of the receptor. An example is ER phosphorylation, which we observed in our models; however, this was not at the high levels previously described in cells transiently transfected with mutant ER [4, 6–8]. Harrod et al. recently reported a Y537S clone generated with CRISPR technology [9], and similarly observed an increase in Ser118 phosphorylation. However, the effect was weaker than estrogen-induced phosphorylation in the WT control cells, and there was no significant difference between WT and mutant cells in response to a drug inhibiting the kinase signaling pathway causing Ser118 phosphorylation. Thus, additional studies are necessary to understand whether there is a causative role of Ser118 phosphorylation in the ESR1 mutant-associated phenotypes.

We observed ligand-independent transcriptional activity of ER in reporter assays, in expression analysis of candidate genes, and in our genome-wide transcriptomic study. Under our experimental conditions, the magnitude of the effect was larger on expression of endogenous candidate genes, such as *PGR* and *GREB1*, compared to effects using the ERE-TK reporter plasmid. This was especially obvious for the D538G mutant in MCF7 cells, where we failed to observe reproducible effects on the ERE-TK reporter, while the same experimental conditions revealed strong induction of endogenous target genes. These data might have implications for the assay design for identification of drugs targeting mutant ER.

The transcriptomic studies identified a number of growth factors and cytokines that were regulated in a ligand-independent manner in the ESR1 mutant cell lines. These included insulin-like growth factor 2 (*IGF2*), a number of wnt ligands, *CXCL12,* and *IL20*. Future studies will address if and how these factors can contribute to ligand-independent growth through autocrine signaling. Of note, the gene expression analysis also revealed novel target genes that were not regulated by E2 in WT control cells. The number of novel genes was significantly higher in the MCF7-Y537S clone described by Harrod et al. [9], and additional studies are necessary to decipher whether these genes are genuine ER target genes, as a result of potential gain-of-function of the mutant receptor.

Our studies show partial resistance of the mutant ER cells to SERMs and SERDs, as measured by IC50 in growth assays. Of note, the magnitude of resistance was dependent on the cell line and mutation site, with Y537S having significantly stronger resistance compared to D538G, similar to that recently reported by Mao et al. [10]. In general, SERDs were more effective than SERMs, with the novel oral SERD AZ9496 having the highest efficacy when comparing the drugs. Supporting the notion of relative SERD efficacy in *ESR1* mutant disease are our mixing experiments in which WT:mutant cells (99:1) do not survive in the presence of fulvestrant, while the mutant cells outgrow the WT cells in the presence of tamoxifen, or in the absence of ligand, in CSS. This is further supported by retrospective analysis of clinical trial samples, recently reported in two independent studies [26, 27]. We have recently opened a trial in which this question will be addressed in a prospective study (NCT02913430).

Finally, we observed significant differences in the effects of mutant ERs between Y537S and D538G, and between T47D and MCF7 cells. For example, fulvestrant-mediated degradation of D538G was less pronounced and E2-induced transcriptional effects and growth response were stronger in D538G, compared to that seen in Y537S. In general, Y537S had stronger endocrine resistance than D538G, in line with clinical data reported from the BOLERO trial in which patients with Y537S mutant tumors had shorter overall survival compared to those with the D538G mutation [28]. Phenotypical differences between the mutants could, at least in part, explain the co-existence of more than one mutation within the same tumor, which has previously been reported [8, 16, 27]. It is important to decipher if and how co-existing *ESR1* mutant-cells interact, and if such interaction provides the tumor with an evolutionary advantage compared to single *ESR1* mutant tumors. It is likely that tumors that represent genetic heterogeneity at the *ESR1* locus may differentially respond to antiestrogen treatments compared to the tumors with a single mutation in the *ESR1* gene. The ultimate goal of the research on ESR1 mutations is to identify treatments that show efficacy in *ESR1* mutant-tumors, and we should expect that such treatment might depend on the specific mutation(s).

These data suggest that there are significant mutation-specific effects that need to be accounted for when determining the effect of mutation on progression in the clinical setting, and potentially in drug development. We also observed cell-line-dependent effects, for example, ligand-independent growth was more obvious in MCF7 compared to T47D cells. Cell-line-dependent effects have previously been described for the study of other mutations [29], and future studies need to address if and how this relates to inter-tumor heterogeneity with respect to the effects of *ESR1* mutation.

## Conclusions

In summary, we have generated robust data in novel experimental model systems representing *ESR1* mutant disease that will facilitate further studies of endocrine resistance. Using biologically appropriate genome-edited models, our comprehensive analysis not only showed that *ESR1* mutants display ligand independent activity, but revealed context-specific and mutation-site-specific features of mutations that should be considered in future studies of *ESR1* mutations.

## Additional files

Additional file 1: Table S1. The sequence of sgRNA and oligos used to generate T47D *ESR1* mutant cell lines via CRISPR. **Table S2** DNA sequence of the oligos used to generate MCF7 *ESR1* mutant cell lines via AAV. **Table S3** Sequence of the primers used for qPCR assay. **Table S4** List of all ligand-independent genes differentially regulated in mutant cells vs WT (FC >2, *p* < 0.005). **Table S5** Disease and function pathways enriched in mutant cells in the absence of estrogen. The novel ligand-independent genes, which were differentially regulated in mutants of each cell line, were pooled and submitted for IPA pathway analysis. The top five relevant functions that were statistically significant are presented in this table. (ZIP 266 kb)

Additional file 2: Figure S1. Sanger sequencing shows the insertion of Y537S (A > C) and D538G (A > G) in T47D and MCF7 cells. **Figure S2** Total ER and phospho ER blotting in all clones of T47D and MCF7 cell lines. **a** Quantification of P-ER(S118) bands from three independent experiments. Band densities were calculated by ImageJ. P-ER values were corrected to total ER level, and then normalized to vehicle-treated WT groups. **b** T47D and MCF7 WT or mutant individual clones were hormone-deprived and treated -/+ 1 nM of E2 for 24 hand IB was performed for ER and p-ER at Ser118 site. B-actin was used as a loading control. **c** Post-hormone-deprived MCF7 or T47D clones were treated with 1 nM of E2 combined with or without 1 μM of Ful for 24 h. RT-qPCR was done using PGR primers. One-way Anova was performed between the basal expression of PGR in each mutant clone and the average expression of PGR in the WT clones (*p < 0.05, **p < 0.01, *red*) and Student’s *t* test was used to compare the response before and after fulvestrant treatment (*p < 0.05, **p < 0.01, *black*). **Figure S3** Lack of significant AR overexpression in MCF7 and T47D *ESR1*-mutant cells: log2 TPM expression of AR in MCF7 and T47D cells based on RNA-seq experiment. **b** The post-hormone-deprived MCF7 or T47D cells (pooled) were treated with 1 nM E2 combined with or without 1 μM of fulvestrant (ICI) for 24 h. RT-qPCR was done using AR-specific primers. **b** Immunoblots of AR expression (CST #5153) in post-hormone-deprived MCF7 or T47D cells. Experiments were performed three times, and AR expression was quantified; *bars* present average AR expression in mutant relative to WT cells. One-way Anova was performed comparing AR mean expression in each mutant clone with mean expression in the WT clones (ns). **Figure S4** The ligand-independent growth of T47D-Y537S clones depends on charcoal-stripped serum (Gibco #12676 serum was used in this experiment). WT or mutant clones were hormone-deprived for 3 days, pooled, and treated with veh or 1 nM E2 for up to 9 days. **Figure S5** Dose–response curves for 2D growth were plotted for Y537S and D538G mutants of T47D (**a**) and MCF7 (**b**) cells after hormone deprivation for 3 days. The cells were treated with 20 pM E2 + Ful, AZD9496, 4OHT and raloxifene. The dose–response curves were fitted with a nonlinear regression model in GraphPad Prism. This figure is a representative of one individual experiment that was repeated six times with consistent results. All experiments were performed in six biological replicates. **Figure S6** PCA analysis of 1000 top variable genes between WT and mutants. The top 1000 most variable genes were selected based on interquartile range. The PCA analysis was performed and plotted using PCA function in R. **Figure S7** Heatmap of variable genes (Anova, *p* < 0.0005, maximum FC >2) in mutants and WT cells. Gene expression TPM was estimated by Salmon package. Anova was then used to identify genes differentially expressed between the samples. Genes with a *p* value <0.0005 and FC >2 that were differentially regulated in at least one mutant vs WT-veh were selected for this heatmap. **Figure S8** The post-hormone-deprived MCF7 or T47D cells (pooled or individual clones) were treated with 1 nM of E2 -/+ 1 μM of Ful for 24 h. RT-qPCR was done using *GREB1* (**a**) or *IGFBP4* (**a**) primers. All experiments were performed in three biological replicates. One-way Anova was performed between the basal expressional levels in each mutant clone and the average expression of *GREB1* and *IGFBP4* in the WT clones (*p < 0.05, **p < 0.01, *red*) and Student’s *t* test was used to compare the response before and after Ful treatment (*p < 0.05, **p < 0.01, *black*). **Figure S9** Log2 TPM expression of *PGR*, *GREB1* and *IGFBP4* levels in MCF7 and T47D cells based on the RNA-seq experiment. **Figure S10.** The post-hormone-deprived MCF7 or T47D cells (pooled or individual clones) were transfected with scramble siRNA or *ESR1* siRNA for 24 h, and then treated -/+ 1 nM of E2 for 24 h. RT-qPCR was done using *ESR1*, *PGR*, or *IGFBP4* primers. All experiments were performed in three biological replicates (one-way Anova, *p < 0.05; **p < 0.01). **Figure S11** Overlap of novel ligand-independent regulated genes of the ESR1 mutations within one cell line (**a**) and between the cell lines (**b**) (chi-square test, **p < 0.01). (PDF 2550 kb)

## Abbreviations

4OHT: 4-Hydroxytamoxifen
AAV: Adeno-associated virus
Anova: Analysis of variance
ATTC: American Type Culture Collection
bp: Base pairs
ChIP: Chromatin-immunoprecipitation
ddPCR: Digital droplet PCR
DMEM: Dulbecco’s modified Eagle’s medium
E2: Estradiol
ER: Estrogen receptor
ERE-TK: Estrogen response element thymidine kinase
FACS: Fluorescence-activated cell sorting
FBS: Fetal bovine serum
FC: fold change
Ful: Fulvestrant
GFP: Green fluorescent protein
IC-50: Half maximal inhibitory concentration
IGF: Inslin-like growth factor
IGFBP: Insulin-like growth factor-binding protein
IL: Interleukin
Greb: Growth Regulation By Estrogen In Breast Cancer 1
PGR: Progesterone receptor
RLU: Relative light units
RNA-seq: RNA sequencing
RPMI: Roswell Park Memorial Institute
SERD: Selective estrogen receptor degraders
SERM: Selective estrogen receptor modulators
siRNA: Small interfering RNA
TPM: Transcripts per million
veh: Vehicle
WT: wild-type
