# Supplementary material for: Mutation site and context dependent effects of ESR1 mutation in genome-edited breast cancer cell models
Source: Breast Cancer Res. 2017 May 23;19:60. doi: 10.1186/s13058-017-0851-4 (PMC5442865; doi:10.1186/s13058-017-0851-4)
Supplement: Supplementary file 1 — The sequence of sgRNA and oligos used to generate T47D ESR1 mutant cell lines via CRISPR. Table S2 DNA sequence of the oligos used to generate MCF7 ESR1 mutant cell lines via AAV. Table S3 Sequence of the primers used for qPCR assay. Table S4 List of all ligand-independent genes differentially regulated in mutant cells vs WT (FC >2, p < 0.005). Table S5 Disease and function pathways enriched in mutant cells in the absence of estrogen. The novel ligand-independent genes, which were differentially regulated in mutants of each cell line, were pooled and submitted for IPA pathway analysis. The top five relevant functions that were statistically significant are presented in this table. (ZIP 266 kb) [file 13058_2017_851_MOESM1_ESM.zip › Suppl. Tables.docx]

**Additional file 1: Table S1-S5**

| **Name** | **Length(bp)** | **Sequence** |
| --- | --- | --- |
| sgRNA targeting ESR1 gene | 20 | TCTCCAGCAGCAGGTCATAG |
| Oligo for Y537S | 70 | GCGGTGGGCGTCCAGCATCTCCAGCAGCAGG TCAGAGAGGGGCACCACGTTCTTGCACTTCATGCTGTAC |
| Oligo for D538G | 70 | GTAGGCGGTGGGCGTCCAGCATCTCCAGCAGCAG GCCATAGAGGGGCACCACGTTCTTGCACTTCATGCT |

**Table S1.**

The sequence of sgRNA and oligos used to generate T47D ESR1 mutant cell lines via CRISPR

| **Homology Arm Cloning Primers** | | |
| --- | --- | --- |
| Targeting Vector | Homology Arm | Forward/Reverse Primer |
| ESR1 exon 10 | 5' | GCAGAGTTGTGGCTAGTGGA/ AAGCTGAGGGCTTTCAGAAG |
|  | 3' | TCCCAGCTCCCATCCTAAAGTG/ AAAGGATGCATTGCCATAGG |
| **Pre-Cre Screening Primers** | | |
| Targeting Vector | Homology Arm | Forward/Reverse Primer |
| ESR1 exon 10 | 5' | GGCAAGTCTCCAACTTGAGC/ GCAGACAGCGAATTAATTCC |
|  | 3' | TTAAGGTACCACTGTGCATATG/ CCGGGAAGATCCAAGTACAG |
| **Post Cre Screening Primers** | | |
| Targeting Vector | Forward/Reverse Primer |  |
| ESR1 exon 10 | GGCAAGTCTCCAACTTGAGC/ CATATGCACAGTGGTACCTTAA |  |
| **Bi-Allelic Sequencing Primers** | | |
| Targeting Vector | Forward/Reverse Primer | Nested Sequencing |
| ESR1 exon 10 | TCCCAGCTCCCATCCTAAAGTG/ AAAGGATGCATTGCCATAGG | CCCCTTCTAGGGATTTCAGC |
| **Targeted Allele Sequencing Primers** | | |
| Targeting Vector | Forward/Reverse Primer | Nested Sequencing |
| ESR1 exon 10 | TTAAGGTACCACTGTGCATATG/ AGAGGCAGAGCTTTCAGCAC | TCCCAGCTCCCATCCTAAAGTG |
| **Mutagenesis Primers** | | |
| Mutation | Forward/Reverse Primer |  |
| ESR1 Y537S | CCTCTCTGACCTGCTGCTGGA/ TCCAGCAGCAGGTCAGAGAGG |  |
| ESR1 D538G | CCTCTATGGCCTGCTGCTGGA/ TCCAGCAGCAGGCCATAGAGG |  |

**Table S2.** DNA sequence of the oligos used used to generate MCF7 ESR1 mutant cell lines via AAV

| **ChIP qPCR** | **Primer name** | **Sequence** |
| --- | --- | --- |
|  | IGFBP4_ChIP+ | GGGTTGGGCAAGGAAAAGTT |
|  | IGFBP4_ChIP- | CTTCTCTGCACCGTGGTTTGT |
|  | GREB1_1_ChIP+ | GTGGCAACTGGGTCATTCTGA |
|  | GREB1_1_ChIP- | CGACCCACAGAAATGAAAAGG |

| **qPCR** | **Primer name** | **Sequence** |
| --- | --- | --- |
|  | RPLP0+ | TAAACCCTGCGTGGCAATC |
|  | RPLP0- | TTGTCTGCTCCCACAATGAAA |
|  | ESR1+ | GAGTATGATCCTACCAGACCCTTC |
|  | ESR1- | CCTGATCATGGAGGGTCAAATC |
|  | IGFBP4+ | ACGAGGACCTCTACATCATCC |
|  | IGFBP4- | GTCCACACACCAGCACTTG |
|  | GREB1+ | GGTTCTTGCCAGATGACAATGG |
|  | GREB1- | CTTGGGTTGAGTGGTCAGTTTC |
|  | PGR+ | TCGCCTTAGAAAGTGCTGTC |
|  | PGR- | GCTTGGCTTTCATTTGGAACG |
|  | AR+ | AATCCCACATCCTGCTCAAG |
|  | AR- | AAGTCCACGCTCACCATG |

**Table S3.** Sequence of the primers used for qPCR assay

**Table S4.** List of all ligand independent genes differentially regulated in mutant cells vs WT (FC>2, p<0.005). See attached excel file (file is too large to be included here).

|  | **Diseases or Functions Annotation** | **p-Value** | **Activation z-score** | **#genes involved** |
| --- | --- | --- | --- | --- |
| T47D | cancer | 5.27E-14 | 0.316 | 394 |
|  | cell movement | 5.50E-14 | 0.446 | 121 |
|  | tumorigenesis of tissue | 2.96E-13 | 1.357 | 375 |
|  | neoplasia of epithelial tissue | 6.30E-13 | 0.951 | 371 |
|  | epithelial cancer | 9.39E-13 | 0.470 | 369 |
| MCF7 | migration of cells | 5.11E-12 | 2.852 | 86 |
|  | cell movement | 2.01E-11 | 2.702 | 91 |
|  | cancer | 1.09E-10 | 0.538 | 290 |
|  | invasion of cells | 1.67E-10 | 1.406 | 49 |
|  | malignant solid tumor | 2.37E-10 | -0.628 | 287 |

**Table S5. Disease and Function pathways enriched in mutant cells in the absence of estrogen.** The novel ligand independent genes, which were differentially regulated in mutants of each cell line, were pooled and submitted for IPA pathway analysis. The top 5 relevant functions that were statistically significant are presented in this table.
