# Supplementary material for: Mutation site and context dependent effects of ESR1 mutation in genome-edited breast cancer cell models
Source: Breast Cancer Res. 2017 May 23;19:60. doi: 10.1186/s13058-017-0851-4 (PMC5442865; doi:10.1186/s13058-017-0851-4)
Supplement: Supplementary file 2 — Sanger sequencing shows the insertion of Y537S (A > C) and D538G (A > G) in T47D and MCF7 cells. Figure S2 Total ER and phospho ER blotting in all clones of T47D and MCF7 cell lines. a Quantification of P-ER(S118) bands from three independent experiments. Band densities were calculated by ImageJ. P-ER values were corrected to total ER level, and then normalized to vehicle-treated WT groups. b T47D and MCF7 WT or mutant individual clones were hormone-deprived and treated -/+ 1 nM of E2 for 24 hand IB was performed for ER and p-ER at Ser118 site. B-actin was used as a loading control. c Post-hormone-deprived MCF7 or T47D clones were treated with 1 nM of E2 combined with or without 1 μM of Ful for 24 h. RT-qPCR was done using PGR primers. One-way Anova was performed between the basal expression of PGR in each mutant clone and the average expression of PGR in the WT clones (*p < 0.05, **p < 0.01, red) and Student’s t test was used to compare the response before and after fulvestrant treatment (*p < 0.05, **p < 0.01, black). Figure S3 Lack of significant AR overexpression in MCF7 and T47D ESR1-mutant cells: log2 TPM expression of AR in MCF7 and T47D cells based on RNA-seq experiment. b The post-hormone-deprived MCF7 or T47D cells (pooled) were treated with 1 nM E2 combined with or without 1 μM of fulvestrant (ICI) for 24 h. RT-qPCR was done using AR-specific primers. b Immunoblots of AR expression (CST #5153) in post-hormone-deprived MCF7 or T47D cells. Experiments were performed three times, and AR expression was quantified; bars present average AR expression in mutant relative to WT cells. One-way Anova was performed comparing AR mean expression in each mutant clone with mean expression in the WT clones (ns). Figure S4 The ligand-independent growth of T47D-Y537S clones depends on charcoal-stripped serum (Gibco #12676 serum was used in this experiment). WT or mutant clones were hormone-deprived for 3 days, pooled, and treated with veh or 1 nM E2 for up to 9 days. Figure [file 13058_2017_851_MOESM2_ESM.pdf]

T47D clones

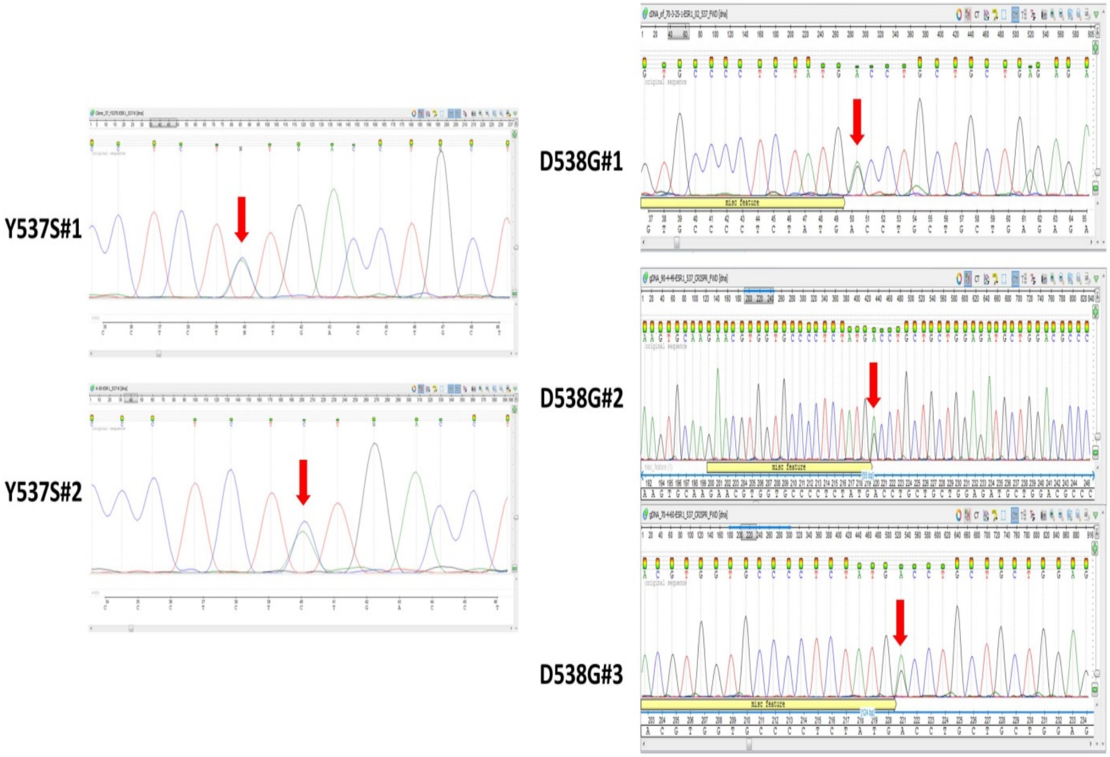

MCF7 clones

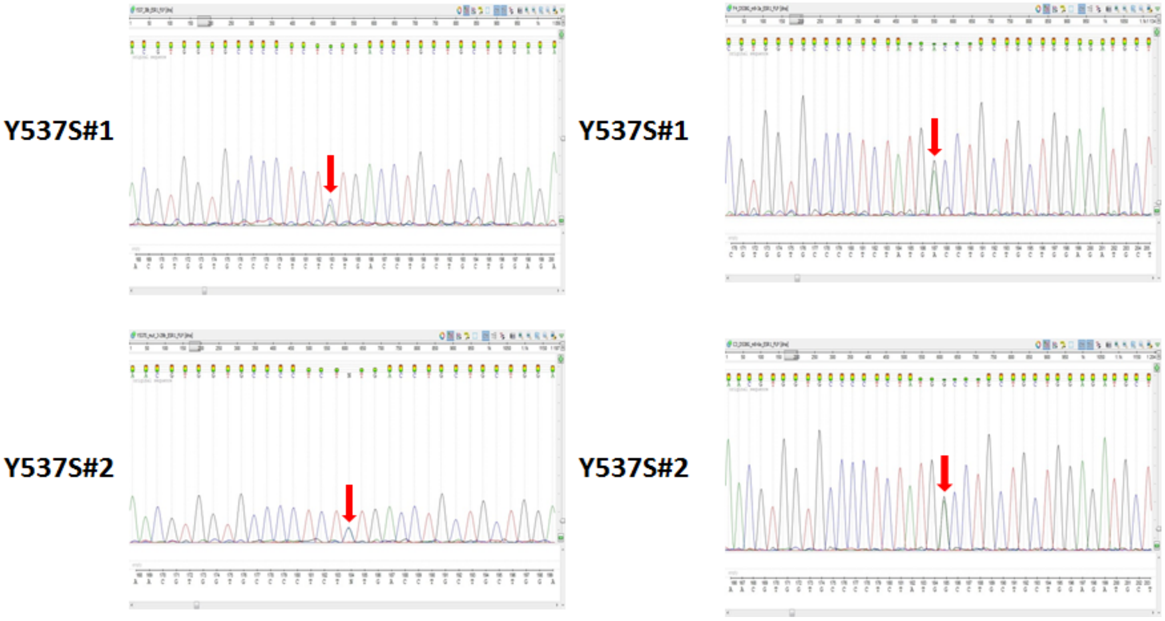

Supplementary Figure S1.

Sanger sequencing of ESR1 mutations in T47D and MCF7 cells.
Sanger sequencing shows the insertion of Y537S (A>C) and D538G (A>G) in T47D and MCF7 cells.

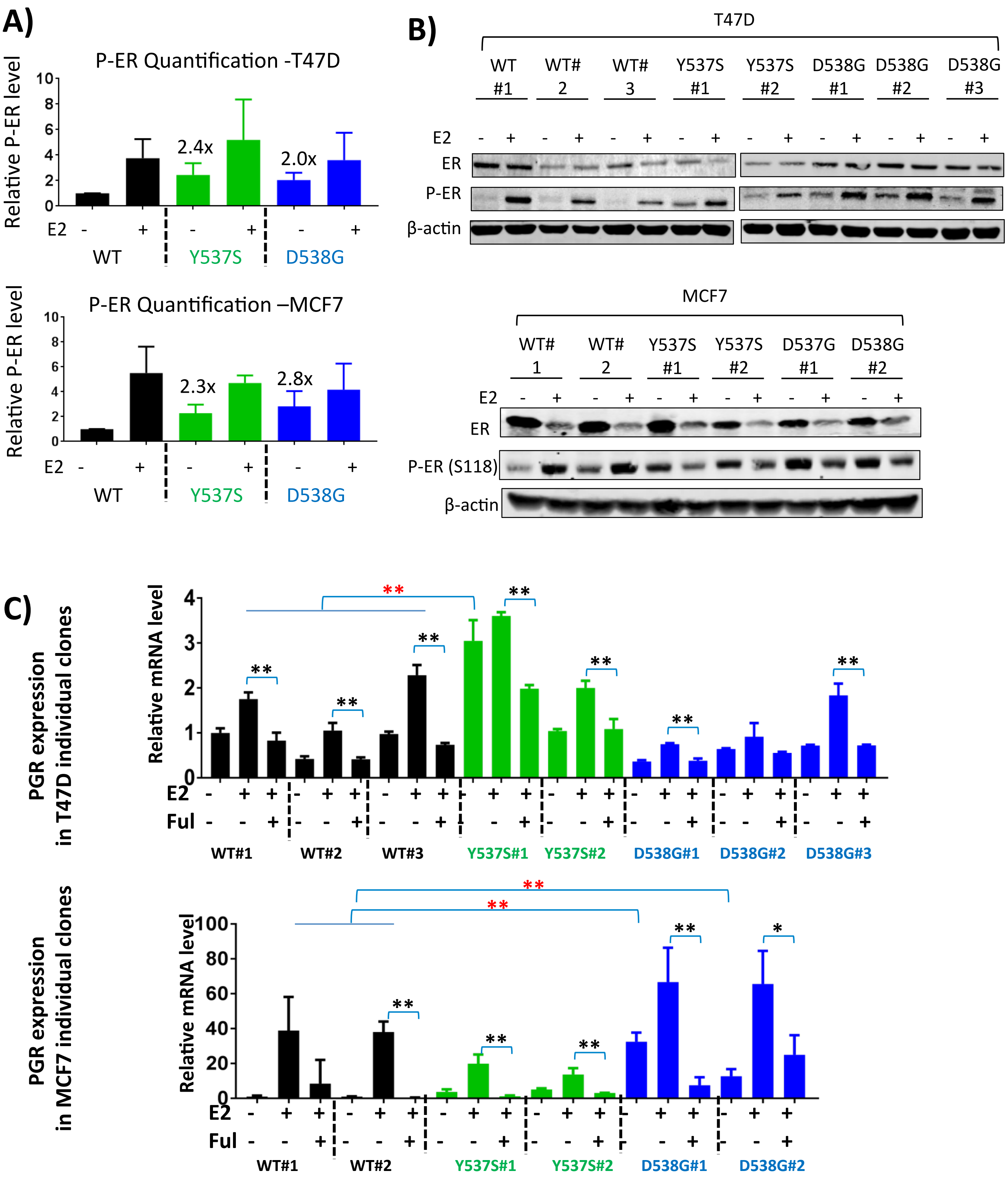

Supplementary Figure S2.

Total ER and phospho ER blotting in all clones of T47D and MCF7 cell lines.

A) Quantification of P-ER(S118) bands from three independent experiments. Bands' densities were calculated by ImageJ. P-ER values were firstly divided to total ER level. The phosphor-portions of each cell line were then normalized to vehicle-treated WT gorups. B) Both T47D and MCF7 WT or mutant individual clones were hormone deprived and treated with or without 1 nM of E2 for 24 hours. The cells were lysed and subjected to western bot detection for ER and p-ER at Ser118 site. B-actin was used as a loading control. C) The post-hormone-deprived MCF7 or T47D individual clones were treated with 1 nM of E2 combined with or without 1 μM of Ful for 24 hours. RT-qPCR was done using PGR primers.

One-way ANOVA was performed between the basal expression of PGR in each mutant clone and the average expression of PGR in the WT clones (* $p<0.05$, ** $p<0.01$, red) and student t-test was used to compare the response before and after fulvestrant treatment (* $p<0.05$, ** $p<0.01$, black).

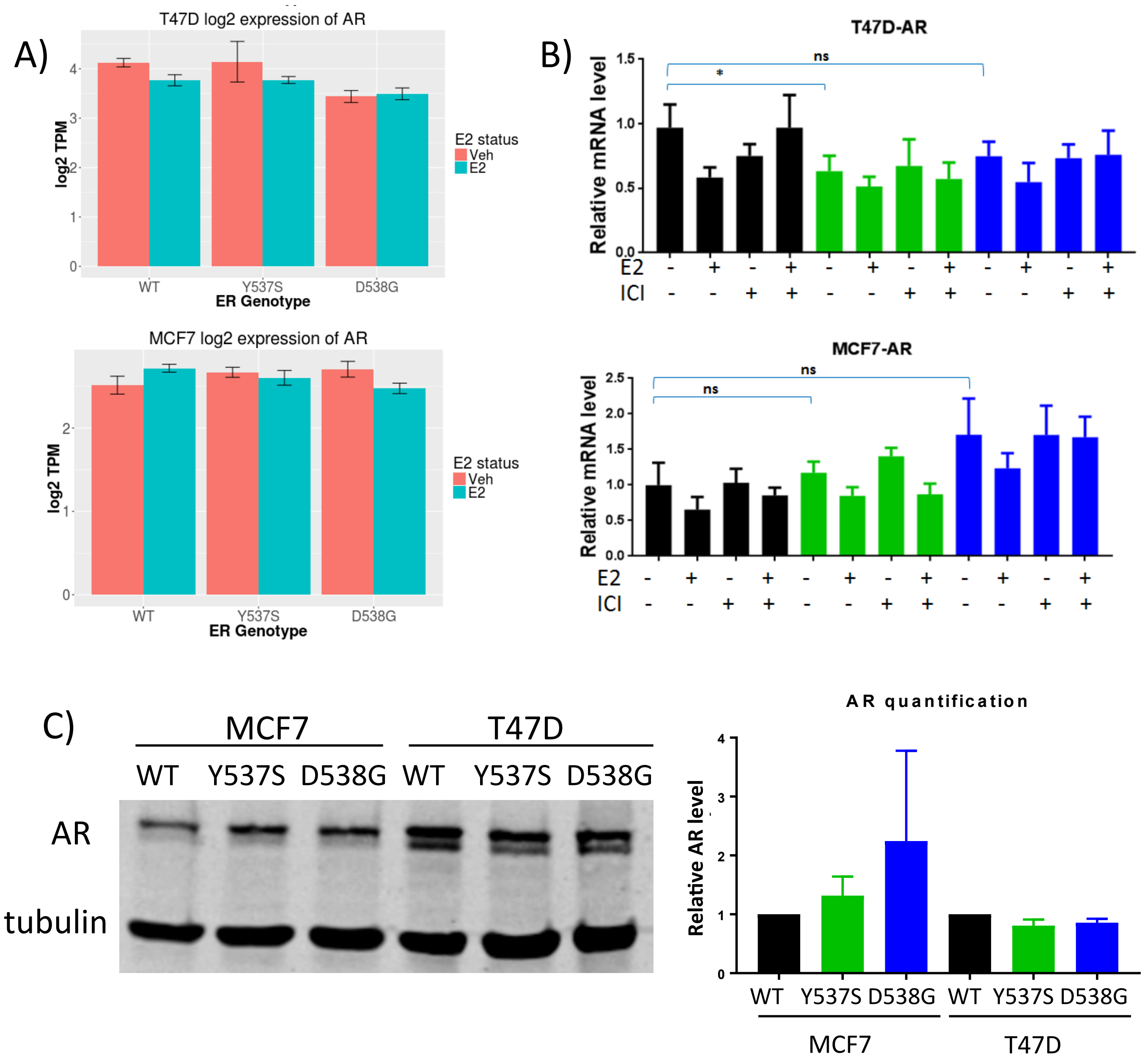

Supplementary Figure S3.

Lack of significant AR overexpression in MCT and T47D ESR1-mutant cells.

A)log2 TPM expression of AR in MCF7 and T47D cells based on RNA-seq experiment. B) The post-hormone-deprived MCF7 or T47D cells (pooled) were treated with 1 nM E2 combined with or without 1 µM of Fulvestrant (ICI) for 24 hours. RT-qPCR was done using AR-specific primers. C) Immunoblots of AR expression in post-hormone-deprived MCF7 or T47D cells. Experiments were performed three times, and AR expression was quantified, and bars present average AR expression in mutant relative to that in WT cells. One-way ANOVA was performed comparing AR mean expression in each mutant clone with mean expression in the WT clones (n.s.).

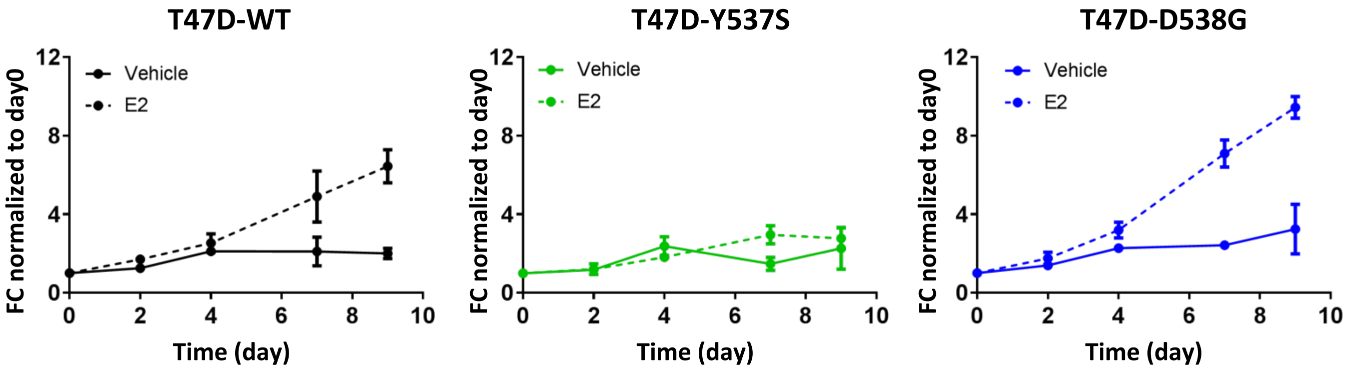

Supplementary Figure S4.

The ligand-independent growth of T47D-Y537S clones depends on charcoal stripped serum (Gibco #12676 serum was used in this experiment).

WT or mutant clones were hormone deprived for 3 days, pooled, and treated with veh or 1 nM E2 for up to 9 days. Cell numbers were quantified by FluoResporter kit.

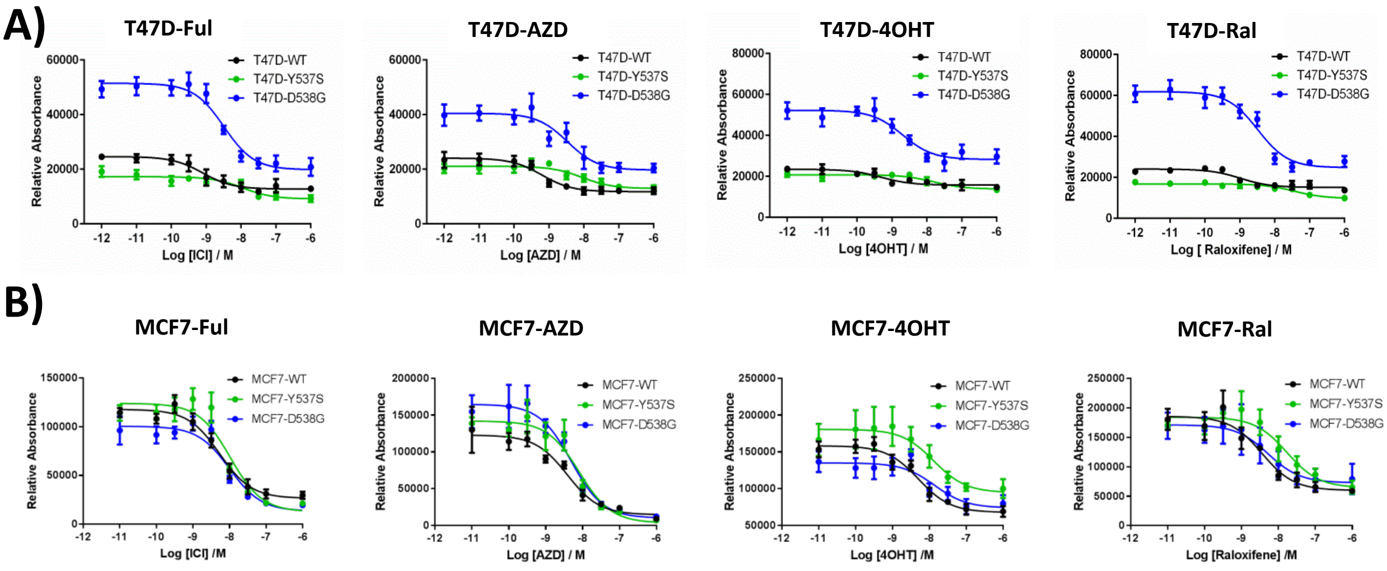

Supplementary Figure S5.

Dose response curves for 2D growth were plotted for Y537S and D538G mutants of T47D **(A)** and MCF7 **(B)** cells after hormone deprivation for 3 days. The cells were treated with 20 pM E2 plus varying doses of Ful, AZD9496, 4OHT and Raloxifene. The dose response curves were fitted with nonlinear regression model in GraphPad Prism. This figure is a representative of one individual experiment that was repeated 6 times with consistent results. All experiments were performed in six biological replicates.

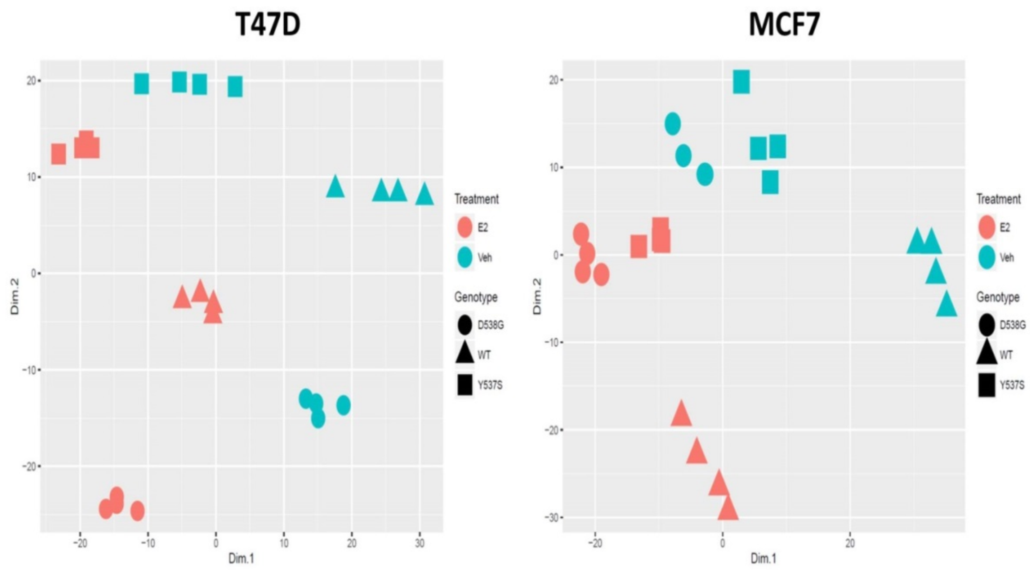

Supplementary Figure S6.

PCA analysis of 1000 top variable genes between WT and mutants.

The top 1000 most variable genes were selected based on interquartile range. The PCA analysis was performed and plotted using PCA function in R.

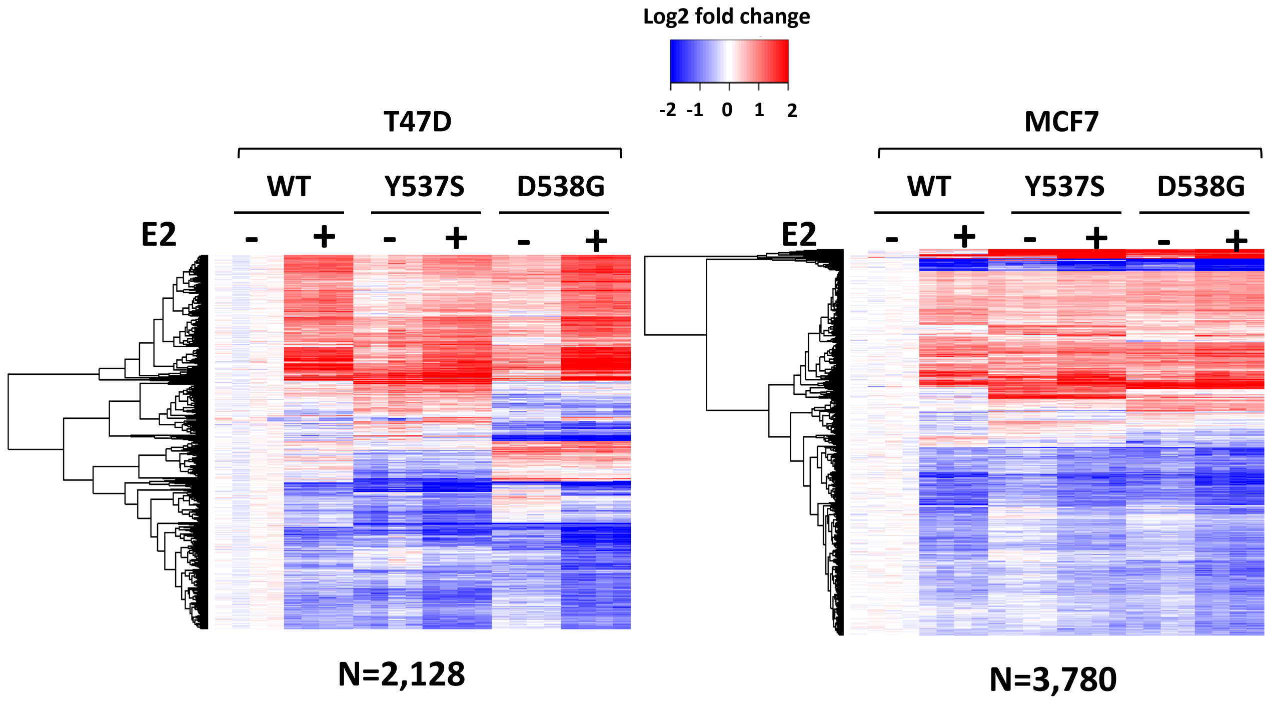

Supplementary Figure S7.

Heatmap of variable genes (ANOVA test=p<0.0005, maxFC>2) in mutants and WT cells. Gene expression TPM was estimated by Salmon package. ANOVA test was then used to identify differentially expressed genes between the samples. Genes with a p<0.0005 and fold change>2 that were differentially regulated in at least one mutant vs WT-veh were selected for this heatmap.

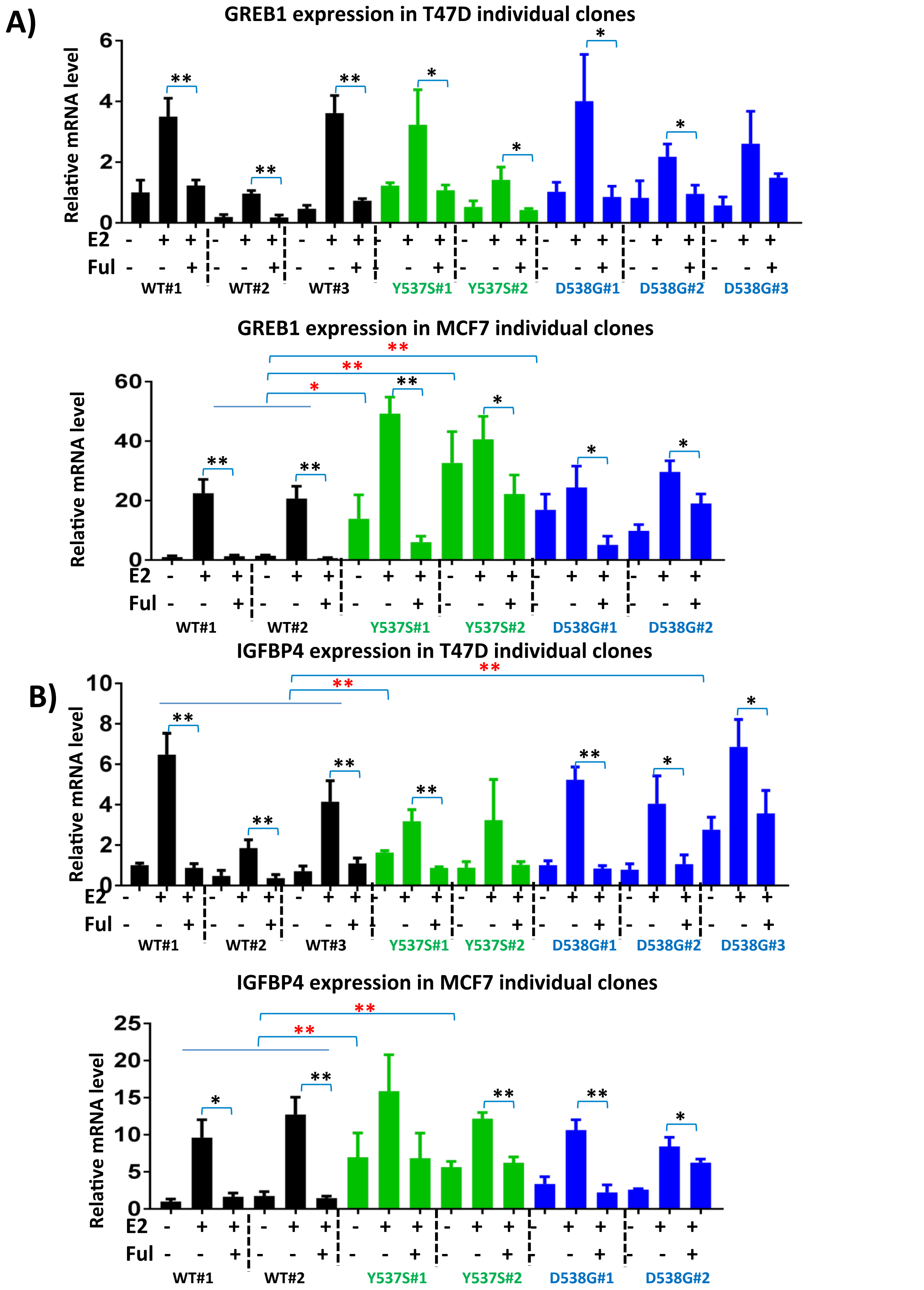

Supplementary Figure S8. The post-hormone-deprived MCF7 or T47D cells (pooled or individual clones) were treated with 1 nM of E2 combined with or without 1 µM of Ful for 24 hours. RT-qPCR was done using GREB1 (A) or IGFBP4 (B) primers. All experiments were performed in three biological replicates. One-way ANOVA was performed between the basal expressional levels in each mutant clones and the average expression of GREB1 and IGFBP4 in the WT clones (* $p<0.05$, ** $p<0.01$, red) and student t-test was used to compare the response before and after fulvestrant treatment (* $p<0.05$, ** $p<0.01$, black).

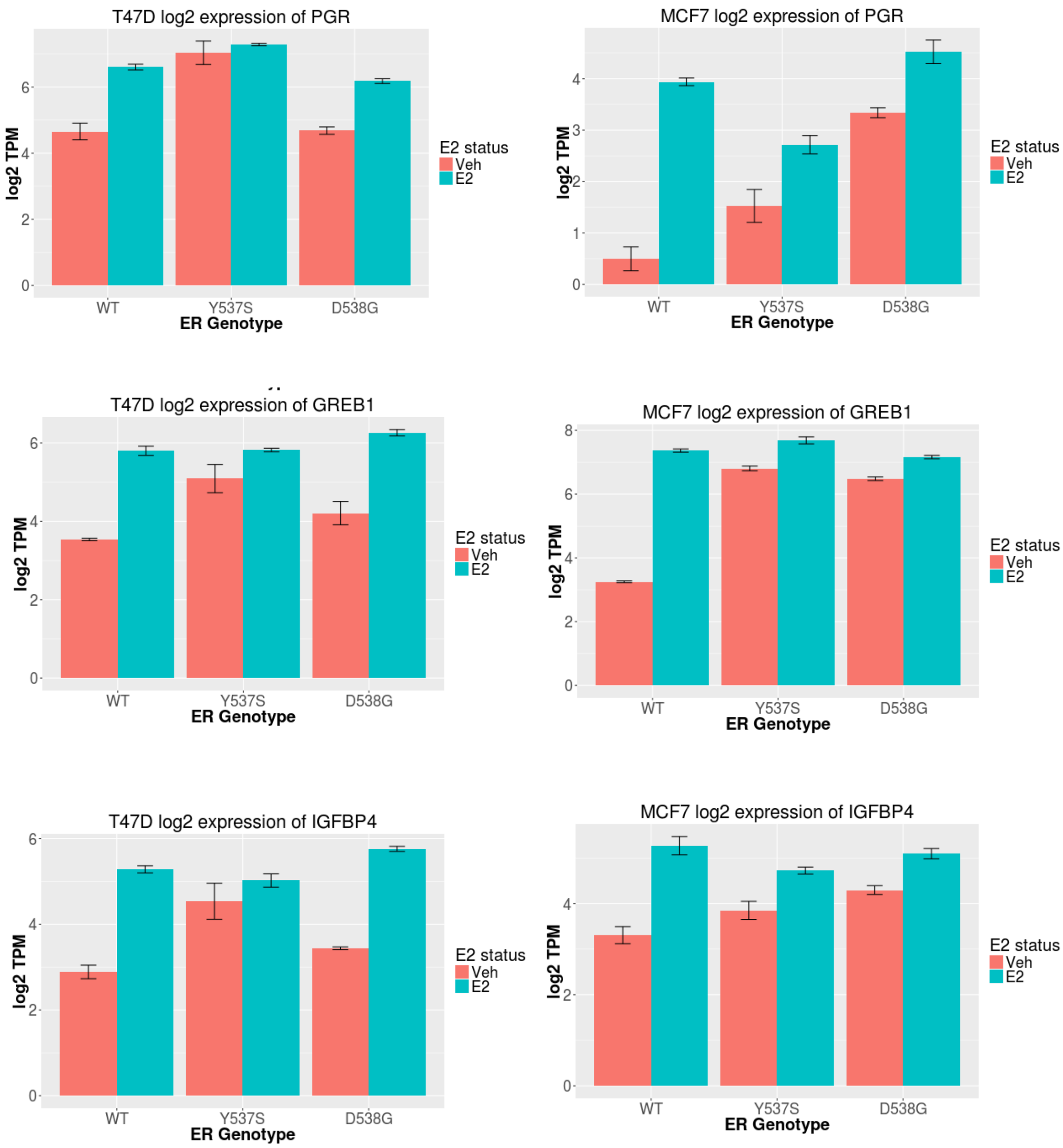

Supplementary Figure S9.
log2 TPM expression of PGR, GREB1 and IGFBP4 levels in MCF7 and T47D cells based on RNA-seq experiment.

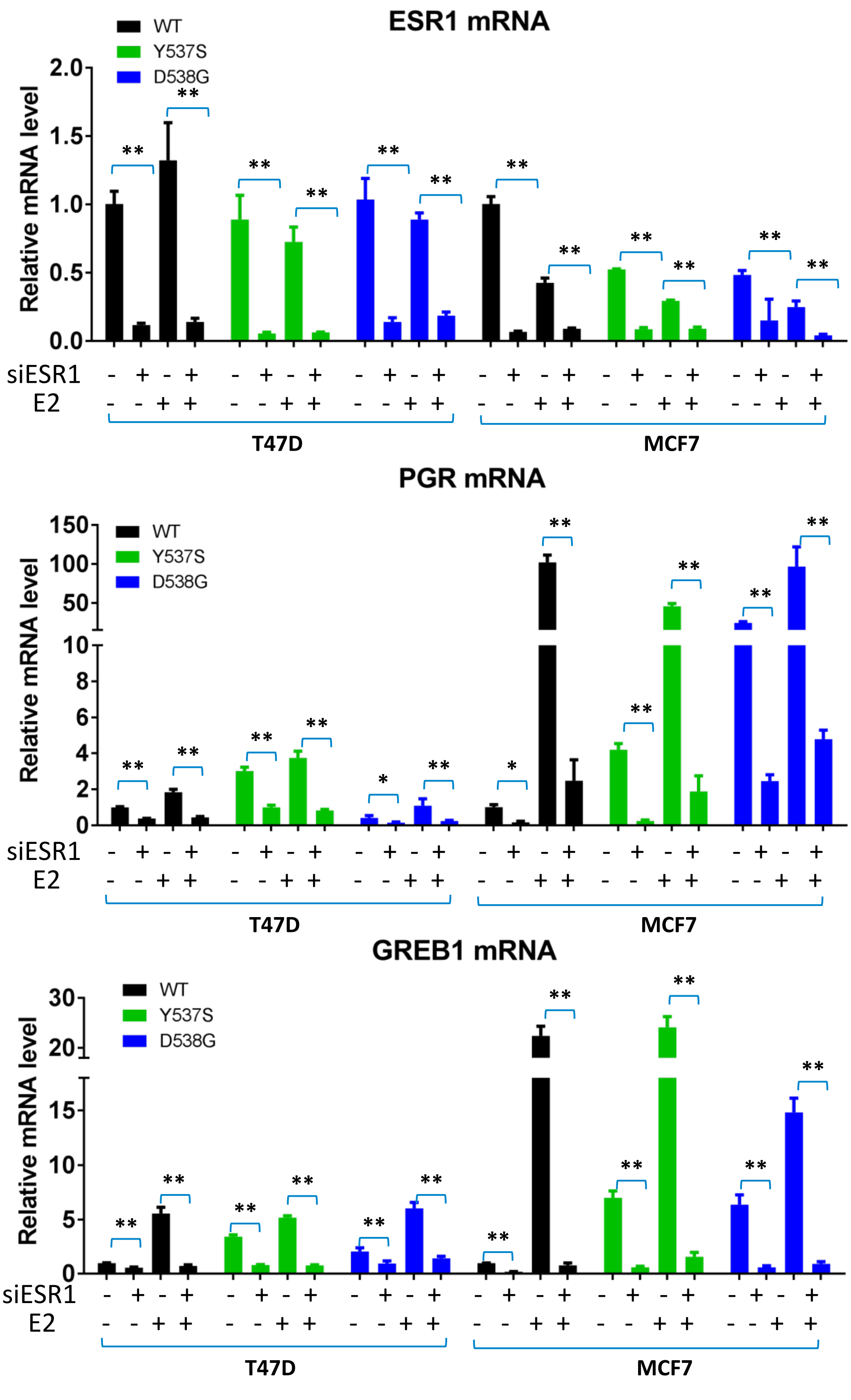

Supplementary Figure S10.

The post-hormone-deprived MCF7 or T47D cells (pooled or individual clones) were transfected with scramble siRNA or ESR1 siRNA for 24 hours, and then treated with or without 1 nM of E2 for 24 hours. RT-qPCR was done using ESR1, PGR or IGFBP4 primers. All experiments were performed in three biological replicates. (one-way Anova, * $p<0.05$; **$p<0.01$)

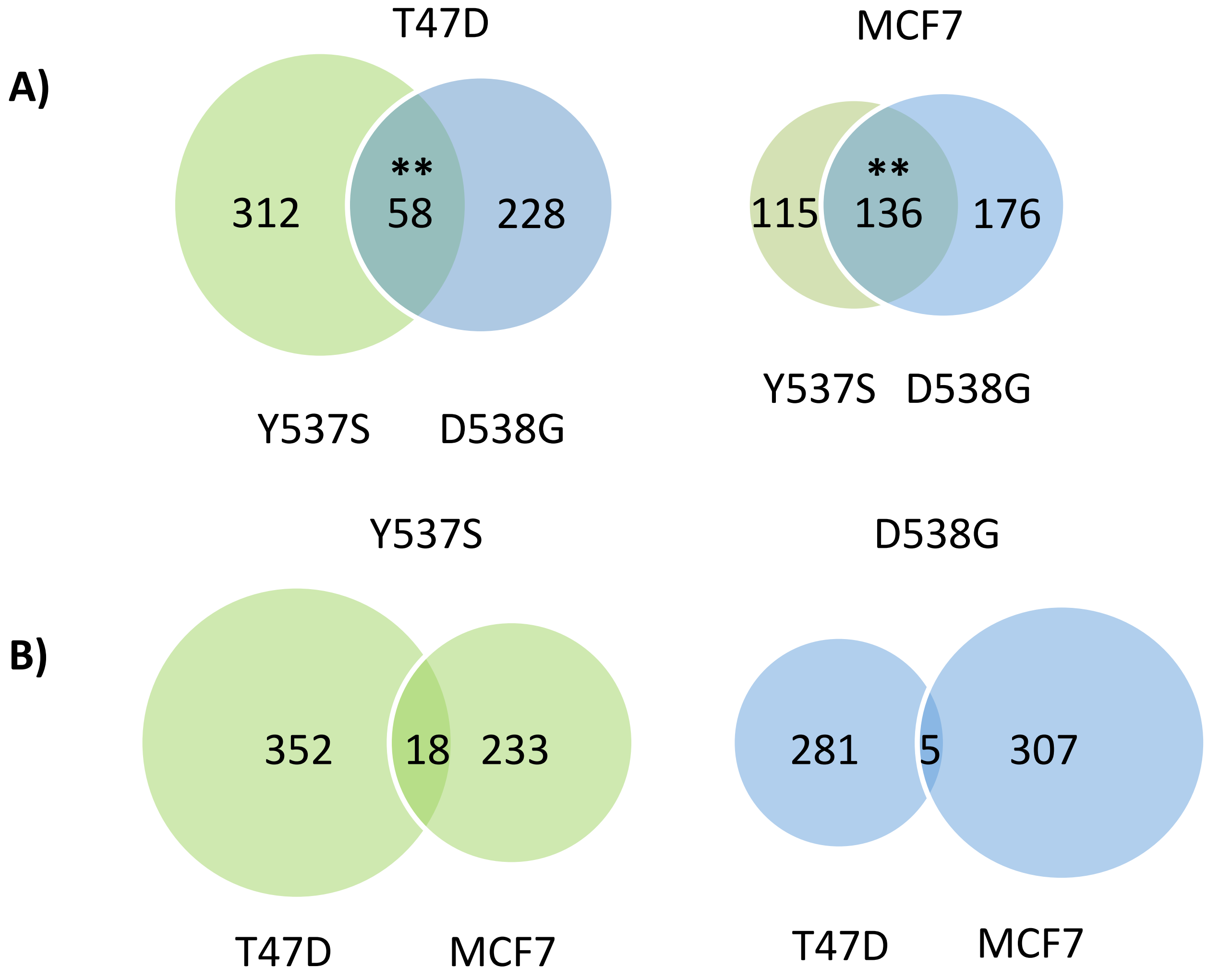

Supplementary Figure S11.
Venn diagram overlap of novel ligand independent regulated genes of the ESR1 mutations within one cell line (A) and between the cell lines (B) (Chi-square test, **$p<0.01$).
